# Supplementary material for: insideOutside: an accessible algorithm for classifying interior and exterior points, with applications in embryology
Source: Biol Open. 2023 Aug 25;12(9):bio060055. doi: 10.1242/bio.060055 (PMC10461464; doi:10.1242/bio.060055)
Supplement: Supplementary information [file biolopen-12-060055-s1.pdf]

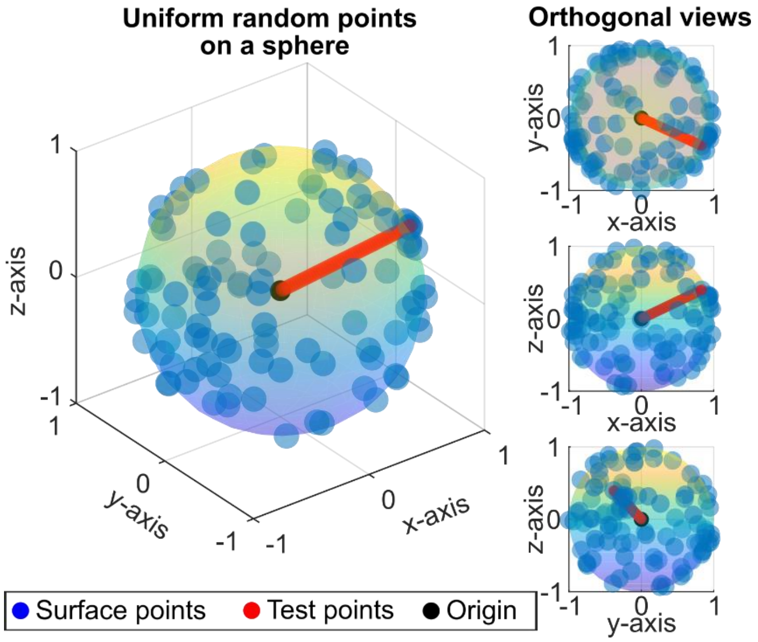

Fig. S1. Example of 100 random points (blue dots) drawn uniformly from the unit sphere (rainbow surface). The minimum distance to the surface, m, and variance in distances to the surface, v, are calculated for 50 test points (red dots) along the vector from the origin (black dot) to a random point on the surface. Shown are the three-quarters view (left) and three orthogonal views (right).

Biology Open • Supplementary information

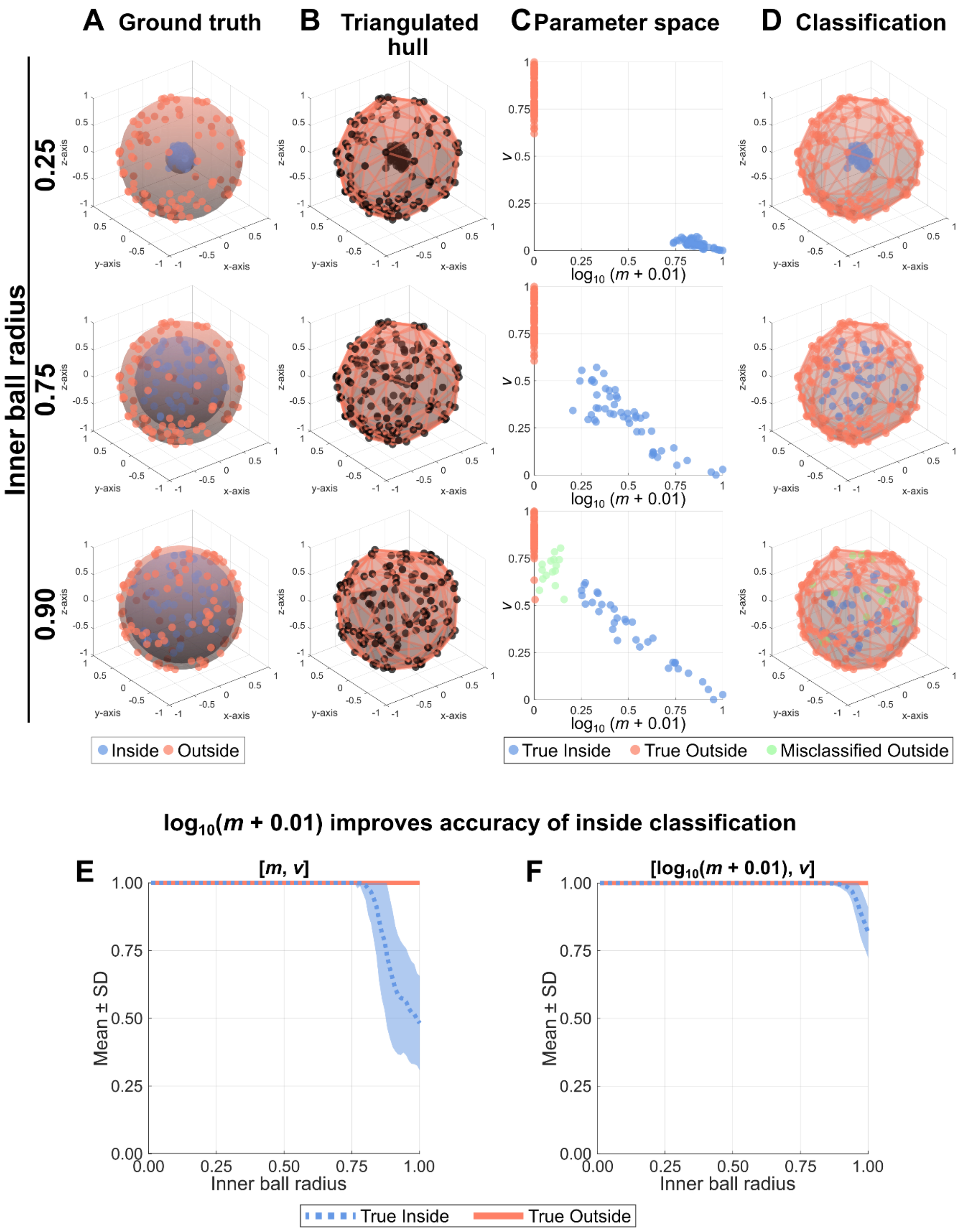

Biology Open • Supplementary information

Fig. S2. Example shapes (50 inside points, 100 outside points) with different inner ball radii used for accuracy testing. **A.** Ground truth of inside points (blue dots enclosed by blue surface) and outside points (orange points on orange surface). **B.** The convex hull generated by the Delaunay triangulation. **C.** Classification of points using hierarchical clustering over the calculated parameter space. Shown are True Inside points (blue), True Outside points (orange), and Misclassified Outside points (green). **D.** The classification mapped onto the original shape. **E-F.** Accuracy testing was performed by classifying the points of 1000 shapes for 100 different inner ball radii. The mean True Inside rate (blue dotted line) is shown with standard deviation (blue filled region) and the mean True Outside rate (orange solid line) is shown with standard deviation (orange filled region). **E.** Accuracy test for the parameters m and v. **F.** Accuracy test for the parameters log10 (m + 0.01) and v.

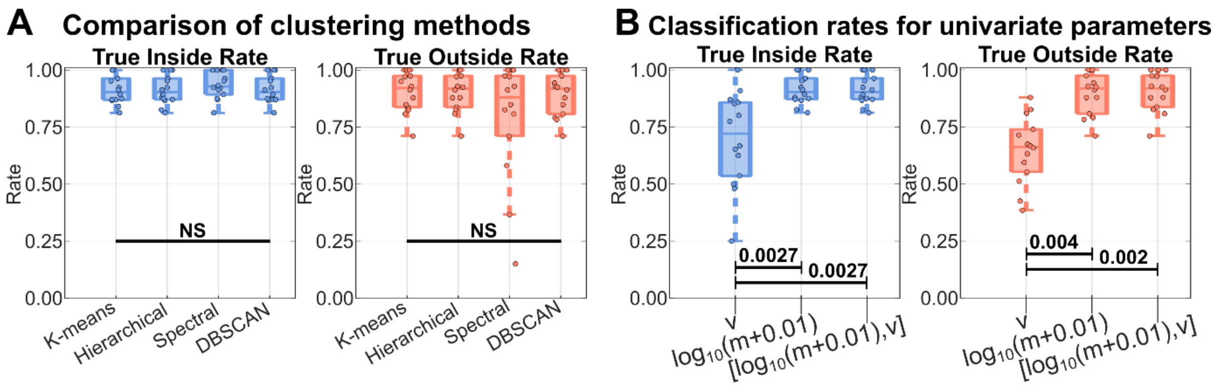

C

	True Inside Rate	True Outside Rate
**Naïve Ellipsoidal**	0.59 ± 0.29	0.73 ± 0.13
**RANSAC Ellipsoidal**	0.62 ± 0.12	0.95 ± 0.06
**Convex Hull**	0.92 ± 0.06	0.89 ± 0.09
**insideOutside**	0.91 ± 0.06	0.90 ± 0.08

Simulated segmentation error

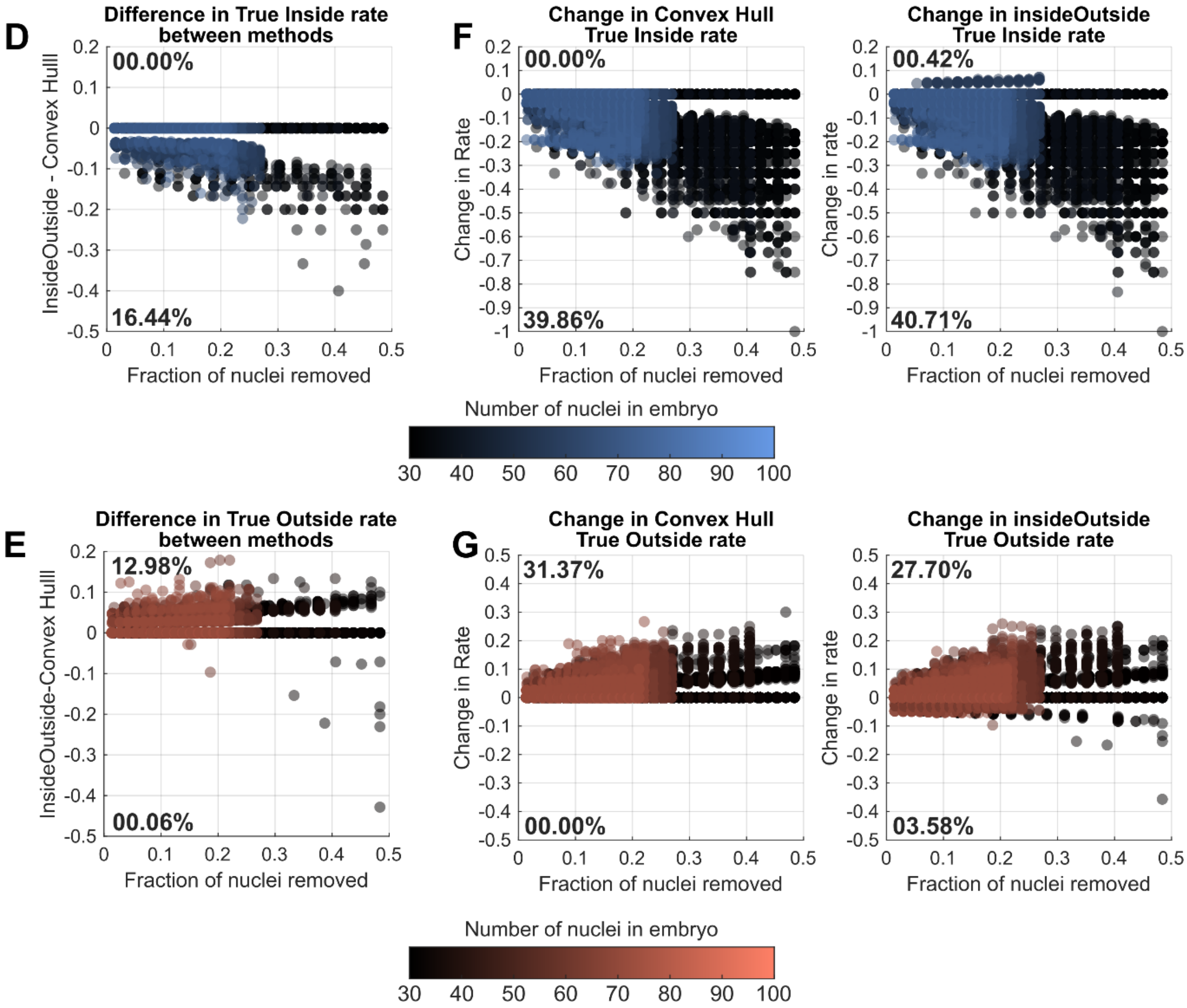

Fig. S3. A. Classification comparison for different unsupervised clustering methods (NS = not significant, p-values > 0.05, Kruskal-Wallis Test). **B.** Classification comparison using a univariate parameter space of mean distance to surface, log10(m + 0.01), or variance in distances to surface, v, or the bivariate parameter space [log10(m + 0.01), v] (p-values, Kruskal-Wallis Test). **C.** Summary of classification rates for different methods. Shown are the mean and standard deviation. **D-G.** Assessing the effects of simulated segmentation error on classification rates. **D-E.** The difference in classification rates between the indieOutside and the Convex Hull Methods for **(D)** inside nuclei and **(E)** outside nuclei. **F-G**. The change in classification rate from the non-subset embryos for indieOutside and the Convex Hull Methods for **(F)** inside nuclei and **(G)** outside nuclei.

Biology Open • Supplementary information

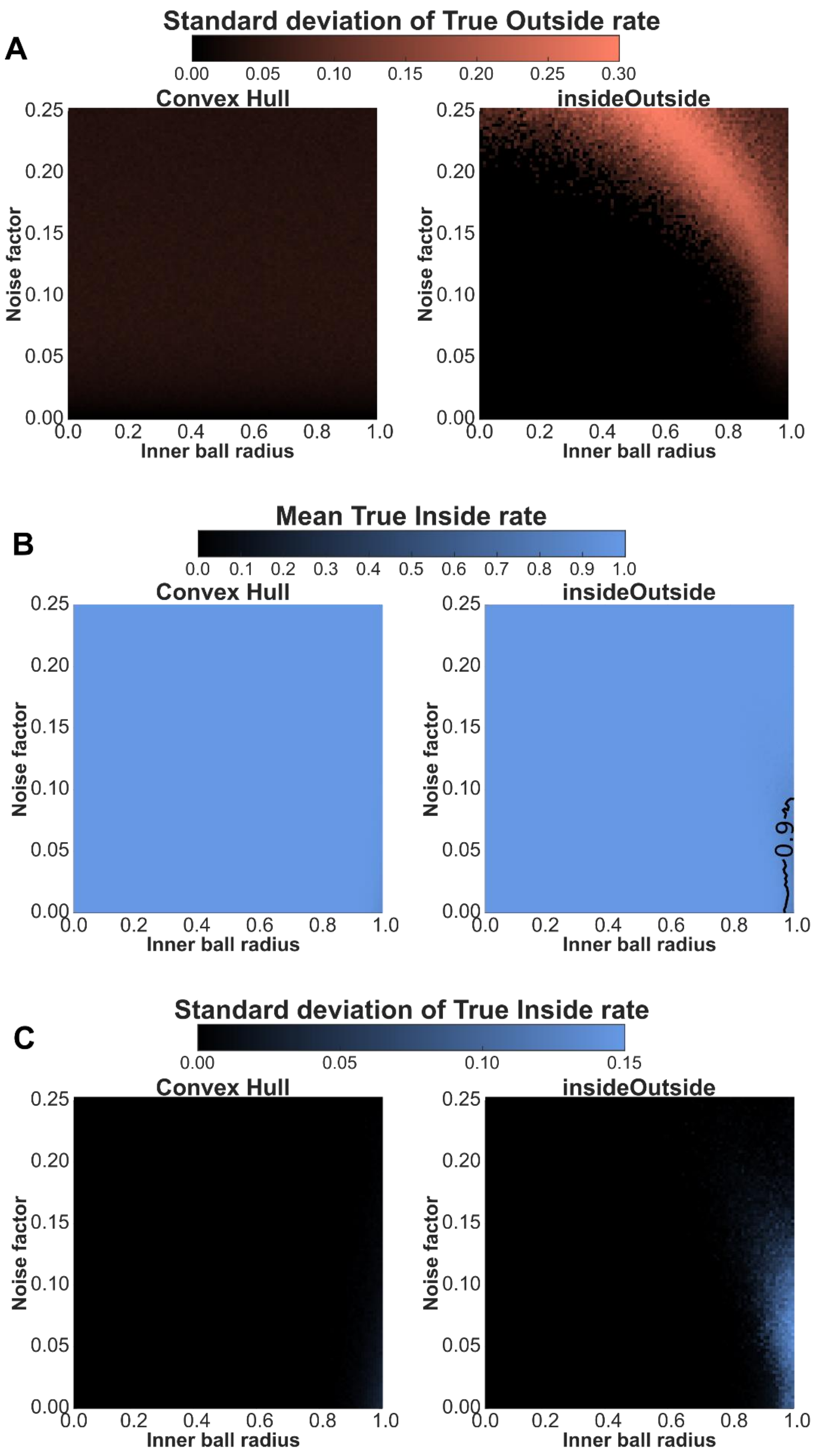

Biology Open • Supplementary information

Fig. S4. Classification rates shown over the parameter space of inner ball radius (100 radii between 0.01 and 1) versus noise factor (100 levels between 0 and 0.25) for the Convex Hull (left) and insideOutside (right) methods. **A.** The standard deviation of the True Outside rate. **B.** The mean True Outside rate. Additional contour lines are shown to delineate drops in classification rate. insideOutside rate is > 0.9 everywhere except when the inner ball radius is close to 1 and the noise factor is small. **C.** The standard deviation of the True Inside rate.
